# 832. OFM Fine Particulate with Split-thickness Skin Graft Supports Functional Healing in Complex Burn Wounds

**DOI:** 10.1093/jbcr/irag033.271

**Published:** 2026-04-06

**Authors:** Michael R Young, Laura K Pezzopane, Olivia Duru, Patrick John Kennedy, Nidhi D Aravapalli, Beth McGuire, Ariel Rodgers, Nicole P Bernal, John H Loftus

**Affiliations:** The Ohio State Comprehensive Burn Center, Columbus, OH; The Ohio State Comprehensive Burn Center, Columbus, OH; The Ohio State Comprehensive Burn Center, Columbus, OH; The Ohio State Comprehensive Burn Center, Columbus, OH; The Ohio State Comprehensive Burn Center, Columbus, OH; The Ohio State Comprehensive Burn Center, Columbus, OH; The Ohio State Comprehensive Burn Center, Columbus, OH; The Ohio State Comprehensive Burn Center, Columbus, OH; The Ohio State Comprehensive Burn Center, Columbus, OH

## Abstract

**Introduction:**

Application of skin grafts for burn reconstruction in areas of high mobility, particularly over joints, presents unique challenges in achieving closure while preserving function. Biologic scaffolds with extracellular matrix proteins may enhance skin graft take and improve wound healing. As these sites are especially susceptible to contracture and graft failure, reconstruction must emphasize both durable wound coverage and preservation of joint mobility.

**Methods:**

A retrospective chart review at a certified comprehensive tertiary burn center was conducted to identify patients with complex thermal injuries that were treated with ovine forestomach matrix (OFM) fine particulate (500-1000 mg) and split thickness skin graft (STSG) as part of closure between January and August 2025. Patient demographics, burn characteristics, graft outcomes, time to closure, complications, and post-operative joint function were recorded.

**Results:**

Three male burn patients, ages 23-65, were identified for inclusion in the case series. All burns had acute full-thickness flame injuries initially treated topically with daily antibiotic topical and nonstick dressings. All presented to our tertiary burn center 14-53 days prior to STSG and OFM fine particulate application. The mean burn size was 473 cm^2^ (range 153–1086 cm^2^). All patients underwent sharp debridement per standard hospital protocol. One patient received a single OFM application 3 days after arrival, followed by OFM fine particulate with STSG 36 days later. Another patient initially received polyurethane temporizing matrix 1 day after arrival but subsequently developed infection and graft loss; OFM fine particulate with STSG was applied 2 days later resulting in complete healing by day fifteen. All three patients achieved 95-100% take at 1 week with full range of motion across affected joints and no complications or graft loss.

**Conclusions:**

OFM fine particulate combined with STSG facilitated rapid closure, high graft take, and preservation of joint function in complex burn wounds. These initial outcomes warrant further exploration in larger studies to validate findings of improved cosmetic and functional outcomes when combining a biologic scaffold with skin grafting in burn repair.

**Applicability of Research to Practice:**

Biologic scaffold–assisted skin grafting using extracellular matrix proteins can improve functional and cosmetic outcomes in challenging burn reconstructions, supporting early mobilization and rehabilitation.

**Funding for the study:**

N/A.

